# Utilization of Water-Insoluble Carbon Nitride-Phosphotungstic Acid Hybrids in Composite Proton Exchange Membranes

**DOI:** 10.3390/membranes14090195

**Published:** 2024-09-13

**Authors:** Xiancan Yuan, Zhongrui Lu, Xiaoyang Jia, Zhuoran Yang, Jian Wang, Xiong Wang, Jun Lin, Shaojian He

**Affiliations:** 1 State Key Laboratory of Alternate Electrical Power System with Renewable Energy Sources, North China Electric Power University, Beijing 102206, China; xiancan@ncepu.edu.cn (X.Y.); luzhongrui@ncepu.edu.cn (Z.L.); jiaxiaoyang@ncepu.edu.cn (X.J.); yangzhuoran@ncepu.edu.cn (Z.Y.); wangjian31791@ncepu.edu.cn (J.W.); 2 South China Institute of Environmental Sciences, Ministry of Ecology and Environment, Guangzhou 510655, China

**Keywords:** proton exchange membrane, sulfonated polyether ether ketone, phosphotungstic acid, carbon nitride, sintering

## Abstract

Phosphotungstic acid (HPW) can retain water in proton exchange membranes to increase proton conductivity; however, its water-soluble nature limits further application. In this work, we combined HPW and graphitic carbon nitride (g-C_3_N_4_) via sintering to prepare water-insoluble hybrids (HWN), where HPW was chemically linked to g-C_3_N_4_ to fix HPW. Then, HWN fillers were added to a sulfonated polyether ether ketone (SPEEK) matrix to prepare composite membranes. The conductivity of the composite membrane with 10 wt% HWN is up to 0.066 S cm^−1^ at room temperature, which is 53% higher than that of the SPEEK control membrane (0.043 S cm^−1^). The composite membrane also showed stable proton conductivity after being immersed in water for 2000 h. Therefore, our study demonstrates that preparing water-insoluble nanofillers containing HPW components through sintering is a promising approach.

## 1. Introduction

The proton exchange membrane (PEM) is one of the core components of proton exchange membrane fuel cells, playing an important role in the operation of proton exchange membrane fuel cells. The ideal proton exchange membrane should possess high proton conductivity, chemical corrosion resistance, and chemical and high-temperature stabilities [1,2,3,4,5,6,7,8]. The performance of proton exchange membranes directly affects the performance of the proton exchange membrane fuel cells [9,10]. Sulfonated polyether ether ketone (SPEEK) has attracted great attention because of its low cost [11,12]. However, the performance of the SPEEK membrane is closely related to the degree of sulfonation; the proton conductivity of the membrane decreases with the decrease in the sulfonation degree, but the mechanical properties increase [13,14]. To maintain the mechanical properties of the membrane while improving the proton conductivity of the membrane, adding inorganic fillers with strong acidity is one of the strategies to improve the proton conductivity [15,16,17].

Heteropoly acid (HPA) has the characteristics of high acidity and proton conductivity [18]. Among them, phosphotungstic acid (H_3_PW_12_O_40_ · nH_2_O, HPW) has strong acidity and excellent proton conductivity and thermal stability, so it is one of the most promising inorganic fillers used in the preparation of composite PEMs [19]. However, HPW has high solubility in water, and the leakage of HPW from the composite membrane leads to the decrease in proton conductivity [20]. Xu et al. [21] doped HPW into SPEEK to prepare composite membranes. However, the HPW loss rate reached 93.5% after soaking in water at 80 °C for 30 days. Therefore, effectively immobilizing HPW in composite membranes is an urgent issue that needs to be addressed. A common method is to combine HPW with inorganic nanomaterials to prepare water-insoluble hybrids. Meng et al. [22]. loaded HPW onto modified rod-like silica (HPW@K-r-SiO_2_) and verified the interaction between HPW and K-r-SiO_2_ through X-ray photoelectron spectroscopy (XPS) and Fourier transform infrared spectroscopy (FTIR). The SPEEK/HPW@K-r-SiO_2_ composite membranes exhibited good performance. Ryu et al. [23]. prepared composite membranes by combining HPW and graphene oxide (GO) and added them to sulfonated poly (arylene ether) (SPAE). The composite membranes showed higher thermal stability than the pristine SPAE membrane, and the size of the composite membranes did not change significantly with the increase in fillers. Peng et al. [24]. grafted imidazole ionic liquid (IMIL) onto graphene oxide (GO) nanosheets to obtain imidazole-functionalized graphene oxide (mGO), and then loaded HPW onto mGO nanosheets through electrostatic interaction, preparing HPW-coupled graphene oxide (HPW@mGO) nanosheets. These HPW@mGO nanosheets were then added to the SPEEK matrix to prepare SPEEK/HPW@mGO nanocomposite membranes. Due to the different proton conduction behaviors of HPW and IMIL, and the long-range two-dimensional (2D) interfacial ion pathways, the proton conductivity of the nanocomposite membranes was significantly improved under various humidity conditions. However, the conductivity decreased after 360 h of immersion, indicating that electrostatic interaction alone may not be sufficient to completely solve the issue of leaching for soluble proton carriers.

Graphitic carbon nitride (g-C_3_N_4_) is a stable two-dimensional layered material analogous to graphene, composed of triazine units interconnected by amino groups [25,26,27]. HPW can interact with the basic groups on the surface of g-C_3_N_4_, similar to the reaction between HPW and ammonium chloride to form ammonium phosphotungstate [28], effectively immobilizing HPW. Additionally, the 2D structure of g-C_3_N_4_ provides a more continuous pathway for proton transfer, which further promotes proton conduction after combination with HPW. Therefore, in this study, we applied a sintering method to prepare HPW/g-C_3_N_4_ hybrids under an air atmosphere, and it was demonstrated that HPW was attached to g-C_3_N_4_ via chemical bonding to form hybrids that are not soluble in water. We then incorporated these hybrids into the SPEEK matrix to prepare the composite membranes and investigated their structure, morphology, and physicochemical properties, providing a novel method to construct more continuous pathways for proton transport in PEM.

## 2. Materials and Methods

### 2.1. Materials

Melamine was purchased from Usolf Company (Qingdao, China). Poly (ether ether ketone) (PEEK) (Victrex 450 PF) was supplied by Victrex (Lancashire, UK). Concentrated sulfuric acid (95–98%) and N’N-dimethylacetamide (DMAC) were purchased from Boenchuangqi Company (Beijing, China). Phosphotungstic acid (H_3_PW_12_O_40_ · nH_2_O, HPW) was purchased from Alfa Aesar (Shanghai, China). Sodium hydroxide (NaOH) was purchased from Innochem Science & Technology Company (Beijing, China).

### 2.2. Preparation of g-C_3_N_4_

**Scheme 1:**
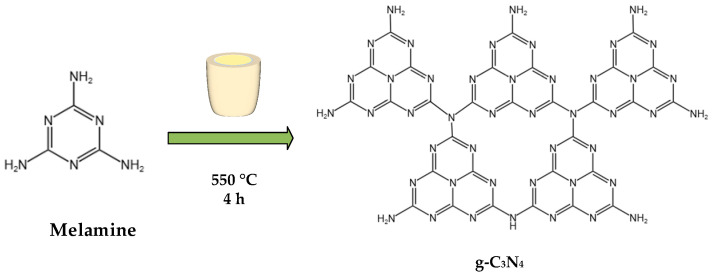
Synthesis of /g-C_3_N_4_.

The preparation of g-C_3_N_4_ was consistent with the literature report [29], as shown in Scheme 1. Melamine (5.0 g) was placed in a crucible sealed with aluminum foil and heated from 25 °C to 550 °C in a muffle furnace at a heating rate of 5 °C min^−1^ and maintained for 4 h. After the furnace was cooled to room temperature, the product named g-C_3_N_4_ was collected.

### 2.3. Preparation of HPW/g-C_3_N_4_

**Scheme 2:**
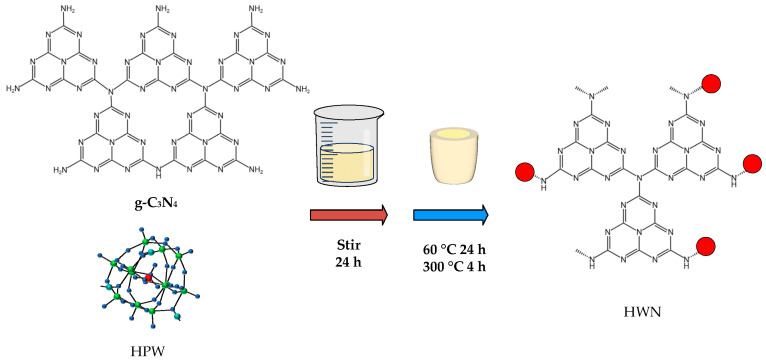
Synthesis of HPW/g-C_3_N_4_.

The specific preparation of HPW/g-C_3_N_4_ is shown in Scheme 2. First, 1.0 g g-C_3_N_4_ was added to 15 mL deionized water for ultrasonic dispersion to obtain a suspension, and then 5.0 g HPW was added to the suspension while stirring, and the stirring was maintained for 4 h. After the mixture was poured into a crucible, it was slowly evaporated and dried at about 60 °C for 24 h, then heated to 300 °C at a heating rate of 5 °C min^−1^ and kept sintering for 4 h. After the furnace was cooled to room temperature, the product was washed three times with deionized water and dried at 60 °C for 24 h to obtain the sintered product of g-C_3_N_4_ and HPW, recorded as HWN.

### 2.4. Preparation of SPEEK

First, 15.0 g PEEK powder was dried in a vacuum oven at 80 °C for 12 h. Next, the dried PEEK powder was added into 300 mL of concentrated sulfuric acid under vigorously stirring at 25 °C for 24 h. Subsequently, the resulting product was poured into ice water to terminate the sulfonation reaction and washed with deionized water repeatedly until the pH reached neutral. The washed product was dried at 40 °C for 12 h followed by 80 °C for 24 h to obtain SPEEK.

### 2.5. Preparation of SPEEK/HWN Composite Membranes

**Scheme 3:**
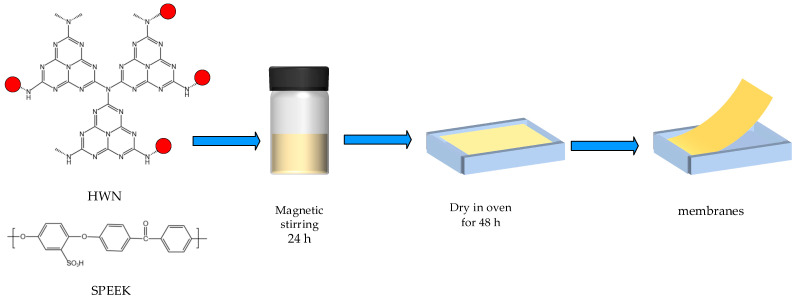
Preparation of SPEEK/HWN composite membranes.

The preparation of composite membrane SPEEK/HWN composite membranes are shown in Scheme 3. A certain amount of HWN hybrid and SPEEK were added to DMAC, maintaining the total of the mass of HWN and SPEEK, which was 0.3 g; when the HWN doping amount was X, the mass of HWN was 0.3 × X, and the mass of SPEEK was 0.3 − 0.3 × X, and it was then stirred for 24 h. Then, the solution was degassed and poured into a glass dish, placed at 80 °C for 24 h, followed by being dried in vacuum at 80 °C for 24 h. The membranes were treated with H_2_SO_4_ of 1.0 mol L^−1^ and washed with deionized water, and the resulting membranes were denoted as SPEEK/HWN-X, where X (X = 2.5, 5, 7.5, 10 and 15) represents the weight percentage (wt%) of HWN hybrids.

### 2.6. Characterization

The X-ray diffraction (XRD) patterns were obtained by a diffractometer (SmartLab SE, Rigaku, Tokyo, Japan). The diffractometer was equipped with a Cu Kα source (λ = 1.54 Å). The scanning rate was 5° min^−1^ and the scanning range was from 5° to 60°. The structure of the powder was characterized by Fourier transform infrared spectroscopy (FTIR) (Nicolet IS10, Thermo Fisher Scientific, Waltham, MA, USA) in the range of 4000–500 cm^−1^. The cross-sectional morphology of the membranes was studied by scanning electron microscope (SEM) (Quattro S, Thermo Scientific, Waltham, MA, USA) at an acceleration voltage of 15 kV. The powder was tested by X-ray photoelectron spectroscopy (XPS) (K-Alpha, Thermo Scientific, Waltham, MA, USA) using Al- Kα source. The powder was analyzed by thermogravimetric analysis (TGA) (TGA 550, TA, New Castle, DE, USA) in nitrogen atmosphere. The temperature range was 30 °C to 800 °C, and the heating rate was 5 °C min^−1^. Stress–strain curves were recorded using a GT-TC 2000 electric tensile tester (Gotech Testing Machine Company, Taiwan, China) according to GB/T 1040, and at least five dumbbell-shaped specimens were tested to give the average.

### 2.7. Water Uptake, Swelling Ratio, Ion-Exchange Capacity (IEC) and Proton Conductivity

The membranes were immersed in deionized water to achieve a fully hydrated state. After 24 h, the membranes were taken out and the water on the surface of the sample was wiped off with filter paper. The weight of the proton exchange membrane is *M_w_*, and the membrane area is *L_w_*. The sample was placed in an oven at 60 °C and dried for 24 h to remove all the water in the proton exchange membrane. The weight at this time was recorded as *M_d_*, and the membrane area was *L_d_*. The formulas for calculating the water absorption and swelling ratio of the membrane are as follows:

(1) $$\mathrm{Water}\;\mathrm{uptake}\left(\%\right)=\frac{M_{w}-M_{d}}{M_{d}}\times 100\%$$

(2) $$\mathrm{swelling}\;\mathrm{ratio}\left(\%\right)=\frac{L_{w}-L_{d}}{L_{d}}\times 100\%$$

The ion exchange capacity (IEC) of the membrane was determined by acid–base titration. In general, the dried sample was immersed in 20 mL NaCl solution (2 mol L^−1^) for 24 h to release H^+^, and then titrated with standard NaOH solution (0.01 mol L^−1^). Using phenolphthalein as an indicator, the calculation formula of IEC was as follows:

(3) $$\mathrm{IEC}=\frac{0.01\times V_{NaOH}}{M_{d}}$$

The proton conductivity (σ) of the membrane was measured by electrochemical workstation (ZENNIUM PRO, Zahner, Kronach, Germany) using AC impedance technique. The membrane samples were first placed in a four-electrode conductivity clamp (BT110, BekkTech LLC, Southern Pines, NC, USA), and then put into liquid deionized water or humidity chamber (YSGDS-50, YISHUO, Shanghai, China) to achieve the desired humidity. The proton conductivity is determined using the following formula:

(4) $$\sigma =\frac{L}{Rdh}$$

where *L* is the distance between electrodes (0.5 cm), *R* is the AC impedance of the membrane, *d* is the width of the membrane (all composite films are around 70 μm), and *h* is the thickness of the proton exchange membrane.

## 3. Results and Discussion

### 3.1. Characterization of HWN

**Figure 1:**
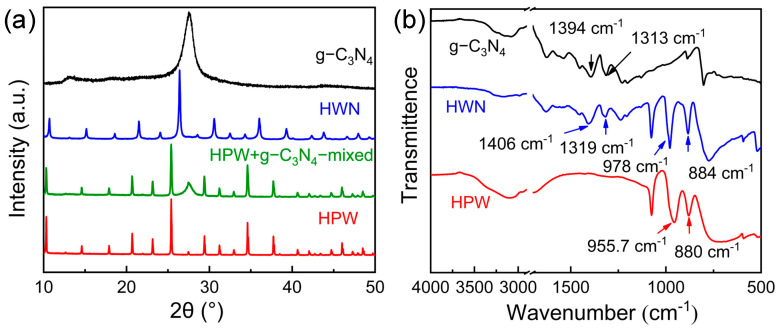
(**a**) XRD patterns of g-C_3_N_4_, HWN, HPW, and g-C_3_N_4_/HPW-mixed; (**b**) FTIR spectra of g-C_3_N_4_, HWN, and HPW.

The crystal structures of g-C_3_N_4_, HPW and sintered product HWN were characterized and analyzed by XRD, as shown in Figure 1a. For g-C_3_N_4_, the XRD pattern demonstrates two major peaks. The characteristic peak at 12.94° corresponds to the (100) crystal plane, which is the in-plane structural stacking of the triazine unit in g-C_3_N_4_, and the characteristic peak at 27.5° corresponds to the (002) crystal plane, which is the inter-plane stacking of the aromatic ring [30]. In the spectra of HPW [31,32], a series of sharp diffraction peaks at 10–40° correspond to the typical characteristic peaks of the Keggin structure [33]. After sintering with HPW, the characteristic peaks of g-C_3_N_4_ in HWN disappear, while the characteristic diffraction peaks of HPW are still maintained, which shows that the Keggin structure was still preserved under high-temperature sintering conditions [34]. However, the characteristic peaks of HWN shift to higher diffraction angles compared with HPW, which indicates that there is a strong interaction between HPW and g-C_3_N_4_. At the same time, we found that the diffraction peak of HWN is highly consistent with the XRD of ammonium phosphotungstate [35]. This indicates that after the sintering reaction, HPW can chemically bond with the amino groups on g-C_3_N_4_ to produce a structure similar to ammonium phosphotungstate. In addition, we compared the mixed g-C_3_N_4_ and HPW with the sintered product. It can be seen from the figure that the characteristic peaks of HPW and g-C_3_N_4_ both appear in the XRD pattern, and no peak shift of the HPW component is observed. The characteristic peak of g-C_3_N_4_ can also be observed, which shows the superposition of the XRD spectra of HPW and g-C_3_N_4_, confirming that HWN is not just a physical mixture of HPW and g-C_3_N_4_.

The chemical state of the fillers is further analyzed by FTIR, as shown in Figure 1b. For g-C_3_N_4_, the peak at 804.5 cm^−1^ is the bending vibration of triazine unit [36], and a series of peaks between 1100 cm^−1^ and 1700 cm^−1^ correspond to the stretching vibration of C-N/C=N [37]. For HPW, the characteristic peaks at 1075 cm^−1^, 958 cm^−1^, 880 cm^−1^, and 760 cm^−1^ are attributed to the stretching vibrations of the P-O_a_ bond of the PO_4_ unit, the stretching vibration of the W=O_d_ bond, and the tensile vibration of the W-O_b_-W and W-O_c_-W bonds of the Keggin element [38,39]. The spectra of HWN between 700 cm^−1^ and 1100 cm^−1^ are almost the same as that of HPW, which indicates that the Keggin structure of HPW is well maintained in HWN. However, the peak position of HWN at 978 cm^−1^ and 884 cm^−1^ is offset compared with HPW, and the peak intensity at 1319 cm^−1^ and 1406 cm^−1^ is offset compared with g-C_3_N_4_, which may be the result of interaction with HPW [40]. Therefore, the FTIR results further proved that there was a new chemical link between HPW and g-C_3_N_4_ after sintering.

**Figure 2:**
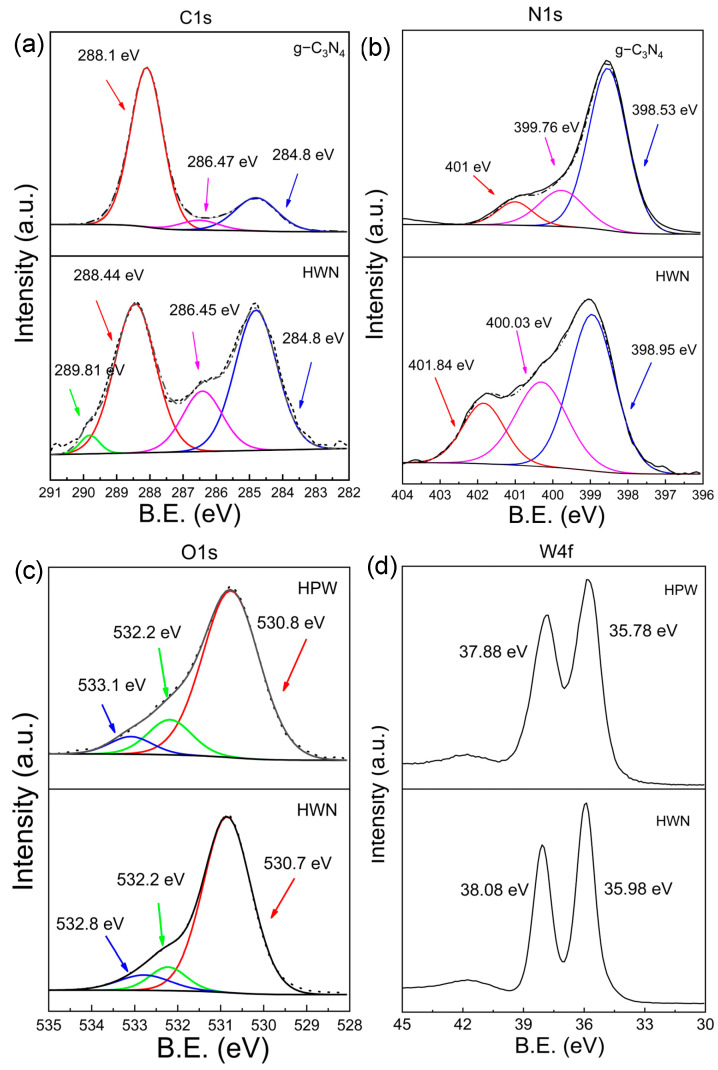
XPS spectra of g-C_3_N_4_ sheets and HWN: (**a**) C1s; (**b**) N1s. XPS spectra of HPW and HWN: (**c**) O1s; (**d**) W4f.

The elemental states of g-C_3_N_4_, HPW and sintered product HWN were further analyzed by XPS. From the C1s spectra (Figure 2a), it can be seen that the peak at 284.8 eV is usually caused by the amorphous carbon in the C-C bond C=C bond on the sample surface [37]. For g-C_3_N_4_, the C1s peak at 288.1 eV comes from N=C (N)_2_ [41], while the C1s peak at 286.47 eV is attributed to the sp2 C atom on the aromatic ring of primary amine and secondary amine (N=C(N)-NH, N=C(N)-NH_2_) [42,43]. For HWN, the peak of C1s shifts to higher binding energy, which may be due to the interaction between g-C_3_N_4_ and HPW [29], and the peak at 289.81 eV may be the result of the formation of C-O-W bond [44]. In the N1s spectra of Figure 2b, the N1s peaks of g-C_3_N_4_ at 398.53 eV and 399.76 eV are attributed to the (C=N-C) aromatic N hybridized by sp2 and the tertiary atom N bonded by (N-(C)_3_), respectively. The N1s peak at 401 eV is the N-H side group [45]. After sintering with HPW, the peak of HWN also shifts to higher binding energy. Compared with g-C_3_N_4_, the peak strength of HWN at 398.95 eV decreases, and the peak strength at 400.03 eV and 401.84 eV increases, which may be caused by the interaction between g-C_3_N_4_ and HPW to generate quaternary ammonium salt cations [29]. In the O1s spectrum. The O1s peak corresponding to W-O-W moves from 530.7 eV of HPW to 530.8 eV of HWN, and the O1s peak of W-O-P moves from 533.1 eV of HPW to 532.8 eV of HWN [46,47]. This means that the O-W bond may interact with g-C_3_N_4_ after the sintering reaction, consistent with the results of C1s. The change in the W4f spectra shown in Figure 2d shows that the peak position of HWN changes from 35.68 eV and 37.78 eV of HPW to 35.98 eV and 38.1 eV, respectively, which further confirms the strong interaction between HPW and g-C_3_N_4_ during sintering.

**Figure 3:**
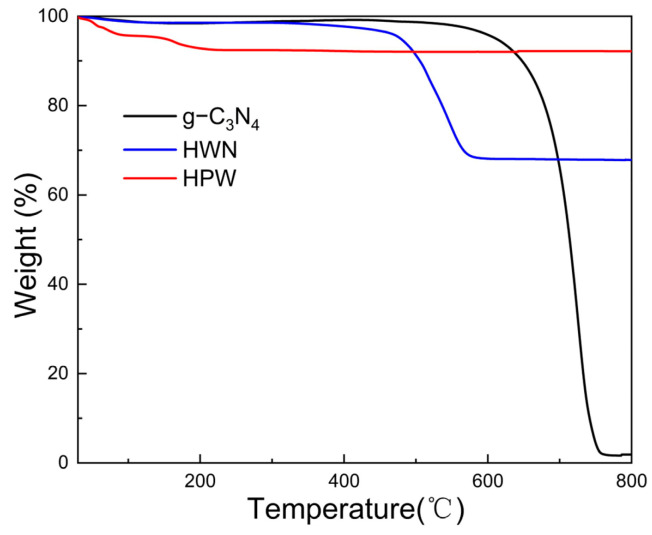
TGA curves of g-C_3_N_4_ sheets, HPW, and HWN.

Thermogravimetric analysis (TGA) was used to analyze the stability of g-C_3_N_4_, HPW, and the sintered product HWN, as shown in Figure 3. The weight loss of g-C_3_N_4_ can be divided into two stages: the first stage is attributed to the loss of surface bound water [48], and the second stage is attributed to the weight loss caused by the decomposition of triazine ring skeleton after 550 °C [49]. It shows that g-C_3_N_4_ has good thermal stability below 550 °C and meets the requirements of fuel cells at high temperature. HPW is very stable below 800 °C, and only weight loss occurs below 200 °C, which is attributed to the evaporation of physically adsorbed water and crystalline water [50]. For HWN, the whole weight loss process is also divided into two stages, the first stage is the loss of bound water on the surface at 30–150 °C, and the second stage is after 450 °C, which may be because the chemical bonds connected to g-C_3_N_4_ and HPW begin to decompose, such as N-H bond breaking from ammonium ions [40]. The weight decomposition of the two stages further shows that the sintering reaction makes g-C_3_N_4_ and HPW chemically linked by chemical bonds, rather than simply physical mixing.

### 3.2. Characterization of the SPEEK/HWN Composite Membranes

**Figure 4:**
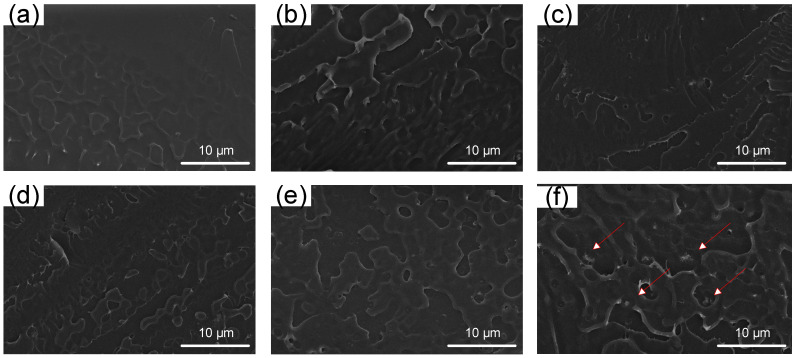
SEM cross-section images of (**a**) SPEEK, (**b**) SPEEK/HWN-2.5, (**c**) SPEEK/HWN-5, (**d**) SPEEK/HWN-7.5, (**e**) SPEEK/HWN-10, and (**f**) SPEEK/HWN-15.

The distribution of HWN in the SPEEK matrix and the microstructure of the composite membranes were observed by SEM, as shown in Figure 4. It can be seen that the SPEEK composite membrane shows a dense morphology, and compared with the SPEEK control membrane, the composite membrane gradually became rough with the increase in HWN doping amount. When the doping amount is less than 10%, the cross-section of the composite membrane is uniform and has no obvious defects, which indicates that HWN can have strong compatibility with SPEEK, which may be due to the strong electrostatic interaction between the phosphotungstate component of HWN and the hydrophilic group of SPEEK. However, when the doping amount is more than 10%, HWN agglomeration can be seen on the surface of the composite membrane.

**Figure 5:**
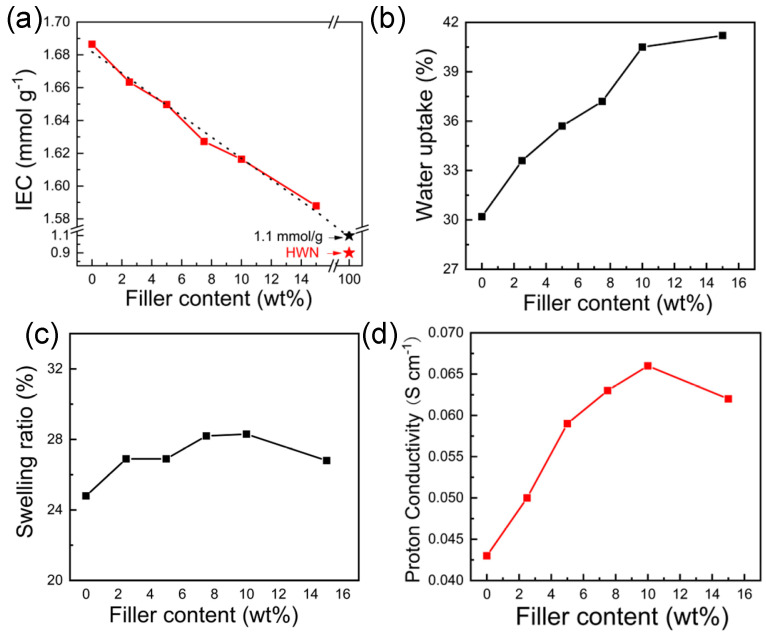
(**a**) IEC, (**b**) water uptake, (**c**) swelling ratio, and (**d**) proton conductivity of SPEEK control and SPEEK/HWN composite membranes in liquid water.

As shown in Figure 5a, the IEC of the composite membrane decreases almost linearly with the increase in filler content, and the decrease may be that the IEC of HWN is lower than that of the SPEEK control membrane. When HWN is added, the basic groups in the filler can interact with the sulfonic acid groups, diluting the -SO_3_H concentration and thus reducing the IEC. However, since HPW provides additional strong acid sites, the dilution is effectively weakened [51,52,53]. In addition, when we linearly fit the IEC to 100% content through the relationship between the IEC and filler content, the IEC of theoretical HWN should be (1.08 mmol g^−1^), which is higher than the result of our titration test (0.09 mmol g^−1^). This shows that NH_2_ on g-C_3_N_4_ can promote the dissociation of sulfonic groups on SPEEK [48,54].

Figure 5b–d shows the relationship between water uptake, swelling ratio, proton conductivity, and filler content of SPEEK/HWN composite membrane. With the increase in HWN doping content, the water uptake of SPEEK/HWN composite membrane increases, which indicates that HWN fillers have strong water absorption capacity. The swelling ratio of SPEEK/HWN composite membranes also changes with the change in water uptake, but generally fluctuates in a very small range, which may be due to the strong interaction between HWN and SPEEK to control the overall stability of the membrane. The conductivity of SPEEK/HWN composite membrane also increases with the increase in HWN doping amount, and the highest conductivity was 0.066 S cm^−1^ when the doping amount reached 10%, which was 53% higher than that of SPEEK control membrane (Figure 5d). The reason for the increase in proton conductivity may be that HPW has strong acidity and HWN can provide a channel for proton transport and reduce the barrier of proton transport. However, when the filler content is more than 10%, the proton conductivity of the membrane decreases, which may be the accumulation of fillers hinders the proton conduction.

**Figure 6:**
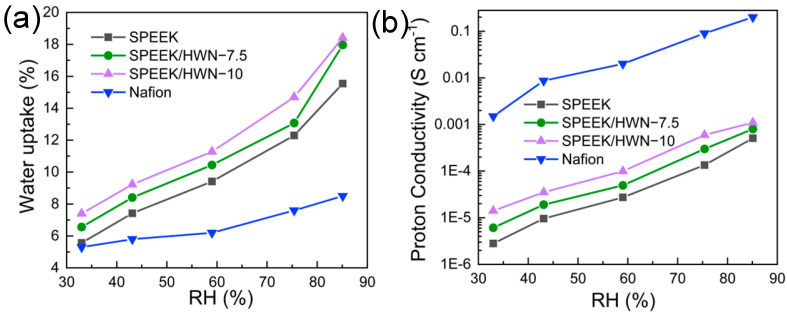
(**a**) Water uptake and (**b**) proton conductivity of Nafion, SPEEK control and SPEEK/HWN composite membranes under low RH.

The water uptake and proton conductivity of SPEEK/HWN composite membranes under different relative humidity (RH) conditions are shown in Figure 6a. At the same RH, the water absorption rate of SPEEK/HWN composite membrane is higher than that of SPEEK control membrane and Nafion membrane, which is mainly due to the strong water retention capacity of HPW component in HWN at low RH, which makes the water retention capacity of SPEEK/HWN composite membrane stronger. The proton exchange membrane will lose a lot of water at low RH, which leads to the decrease in membrane conductivity. SPEEK/HWN composite membranes has a strong water retention capacity, which is more conducive to its proton transport at low RH. As can be seen from Figure 6b, when the RH is less than 80%, the conductivity of the composite membranes is almost an order of magnitude higher than the SPEEK control membrane.

As shown in Table 1 and Figure 6b, it can be seen that at 25 °C and low RH, there is a certain gap between the proton conductivity of the SPEEK/HWN-10 composite membrane and Nafion 212 membrane. According to the proton conductivity improvement effect of the HWN filler in this study, when HWN is added to the SPEEK matrix with higher conductivity, it can reach an order of magnitude of Nafion membrane.

**Figure 7:**
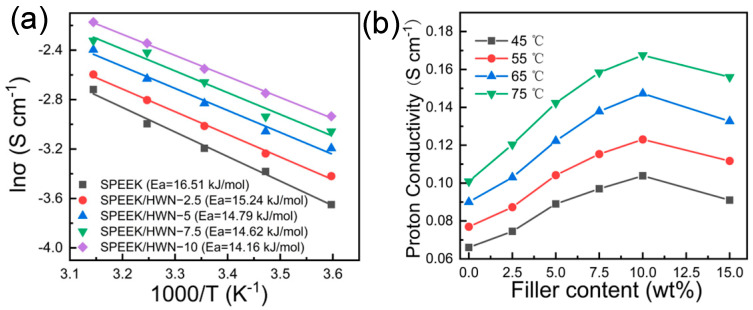
(**a**) Arrhenius plots of proton conductivity as a function of temperature for SPEEK control and SPEEK/HWN composite membranes and (**b**) proton conductivity of SPEEK control membrane and composite membranes at 45–75 °C.

To further explain the proton transport mechanism of HWN in the composite membranes, the conductivity of the composite membranes in liquid water at different temperatures was measured, and the activation energy (*Ea*) of proton transfer of the composite membranes was calculated by the Arrhenius equation [55,56]. As shown in Figure 7a, the *Ea* of the composite membranes decreases gradually with the increase in HWN doping. In general, the *Ea* of SPEEK/HWN composite membrane and its SPEEK control membrane range from 14 to 40 kJ/mol, indicating that the proton transport in the membranes are dominated by the Grotthus mechanism, and protons jump between proton conductors in the membranes through hydrogen bonds, indicating that doping HWN can provide a proton transport channel and reduce the proton transport barrier in the composite membrane [57]. At the same time, we also tested the conductivity of SPEEK control and composite membranes at 45–75 °C. As shown in Figure 7b, as the temperature increases, the proton conductivity gradually increases and the maximum conductivity can reach 0.17 S cm^−^^1^, which indicates that the composite membrane operates normally at 75 °C.

**Figure 8:**
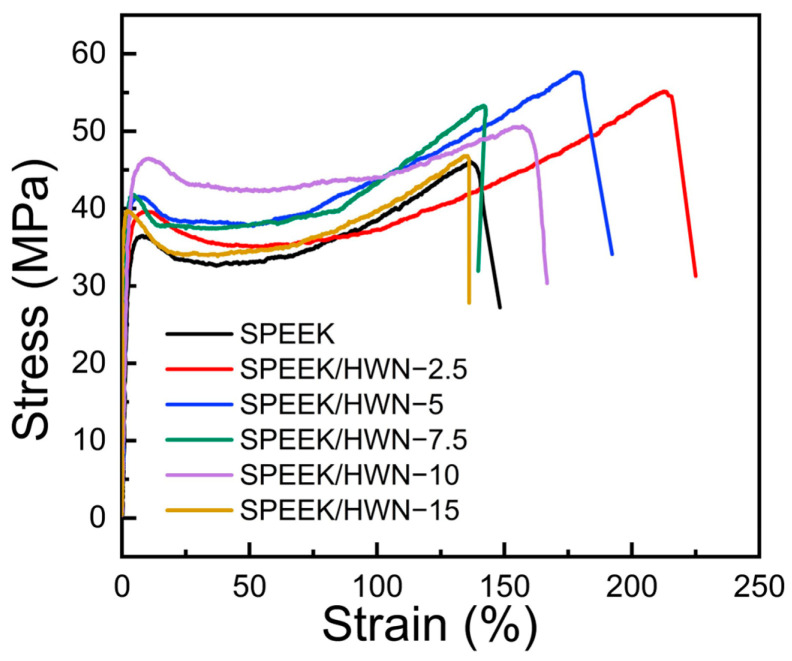
Stress-strain curves of SPEEK control and SPEEK/HWN composite membranes.

In addition, to further analyze the mechanical stability of the membrane, the tensile strength and elongation at break of the composite membranes were tested. As shown in Figure 8, the tensile strength and elongation at break of the SPEEK control membrane are 46.1 MPa and 136%, respectively. For the SPEEK/HWN composite membranes, its tensile strength and elongation at break are higher than those of the SPEEK control membrane. The tensile strength of the SPEEK/HWN-5 membrane is the highest (57.6 MPa), which is much higher than the commercial Nafion 212 membrane (16.1 MPa) [58]. The elongation at the break of the SPEEK/HWN-2.5 membrane is the largest, at 213%. The improvement in the mechanical strength and toughness of the composite membranes can be attributed to the interaction between the HWN and SPEEK matrix.

**Figure 9:**
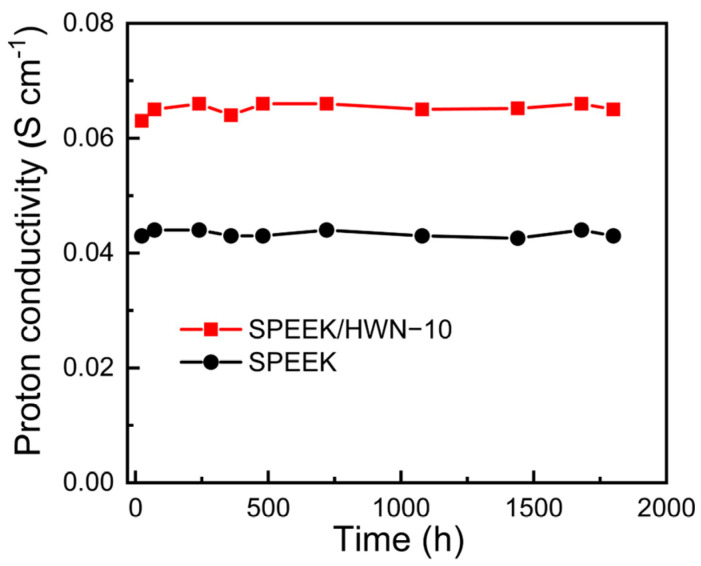
Proton conductivity of the SPEEK control and SPEEK/HWN-10 composite membrane at various times during the water immersion test at 25 °C.

The prepared SPEEK/HWN composite membrane was immersed in liquid water at 25 °C, and the conductivity for nearly 2000 h was obtained, as shown in Figure 9. The proton conductivity of the SPEEK/HWN composite membrane remained unchanged after three months, indicating that most of the HWN particles were retained in the composite membranes. At the same time, a small amount (5 mL) of the liquid water was sampled for UV-Vis analysis to detect the concentration of HPW in water. As the detection limit of our UV-Vis instrument can reach as low as 1 mg L^−1^, this means that after this immersion period, the HPW in the water was less than 0.05 mg, which is less than 1/900 of the maximum weight of HWN doping. Therefore, we can conclude that no HPW was leached from these HWN hybrids. This fully demonstrates that the amino group on g-C_3_N_4_ combines with HPW to form a stable structure, thus immobilizing HPW and ensuring the stability of proton conductivity of the composite membranes.

## 4. Conclusions

In this work, HPW and g-C_3_N_4_ were combined by sintering reaction to prepare water-insoluble HWN hybrids. The structural characterization of XRD, FTIR, and XPS showed that HPW was chemically bonded to g-C_3_N_4_ after the sintering reaction. Due to the relatively strong electrostatic interaction between HWN and sulfonic groups in the SPEEK matrix, the excellent interfacial compatibility of SPEEK/HWN composite membranes was observed by SEM. This interaction makes the water uptake and swelling rate of the composite membrane fluctuate in a very small range. With the additional help of the strong acidity of HPW, the proton conductivity of the membranes increased significantly, and the proton conductivity of SPEEK/HWN-10 membrane reached 0.066 S cm^−1^, which was 53% higher than that of SPEEK control membrane. When the relative humidity is lower than 80%, the proton conductivity of SPEEK/HWN-10 membrane is one order of magnitude higher than that of the SPEEK control membrane. In addition, after nearly three months of liquid water immersion, the proton conductivity of the composite membrane did not decrease significantly. Therefore, this work provides another simple and promising method for preparing water-insoluble solid proton conductors from HPW components, which can be further applied to the fabrication of high-performance nanocomposite proton exchange membranes.

## Figures and Tables

**Table 1:** Water uptake, swelling ratio and IEC of Nafion212 membrane, SPEEK control membrane, and composite membranes at 25 °C.

Membrane	IEC (mmol g^−1^)	Proton Conductivity (S cm^−1^)	Water Uptake (%)	Swelling Ratio (%)
SPEEK	1.680	0.043	30.2	24.8
SPEEK/HWN-10	1.616	0.066	40.5	28.3
Nafion212	0.890	0.100	22.0	16.0

## Data Availability

The original contributions presented in the study are included in the article, further inquiries can be directed to the corresponding authors.
